# Two-dimensional Fibonacci grating for far-field super-resolution imaging

**DOI:** 10.1038/srep38651

**Published:** 2016-12-09

**Authors:** Kedi Wu, Guo Ping Wang

**Affiliations:** 1 College of Electronic Science and Technology and Guangdong Provincial Key Laboratory of Optoelectronic Micro/Nano Optomechatronics Engineering, Shenzhen University, Shenzhen 518060, China

## Abstract

A two-dimensional (2D) Fibonacci grating is used to transform evanescent waves into propagating waves for far-field super-resolution imaging. By detecting far-field intensity distributions of light field through objects in front of the 2D Fibonacci grating in free space at once, we can retrieve the image of objects with beyond λ/7 spatial resolution. We also find that the coherent illumination case can give a better resolution than incoherent illumination case by such 2D grating-assisted imaging system. The analytical results are verified by numerical simulation.

Optical microscopy is a most popular real-time imaging tool in wide applications of life science and nanotechnology^1^. However, the far-field spatial resolution of a standard microscopy is restricted to the wavelength of illumination light (*λ*) and the numerical aperture (NA) of the objective lens, because this kind of imaging system is diffraction limited^2^. The spatial resolution characterizes the minimum resolvable capability of the imaging system. It is generally acknowledged as 0.61*λ*/NA in two-dimensional (2D) system or 0.5*λ*/NA in one-dimensional (1D) system^1^,^2^. To get the super-resolution images of objects, we need to enhance the spatial resolution for collecting the high spatial frequency information of objects. Such information is carried by the evanescent waves, which are only bounded and detectable in the near-field area. The near-field scanning optical microscopy can be used to capture evanescent waves of objects and further to image the subwavelength details of objects^3^. However, there is a time-consuming point-by-point scanning process by approaching a probe to the surface of objects in the near field. Structured light illumination is also a well-established technique to enhance the spatial resolution^4^,^5^,^6^,^7^,^8^. In this case, the object is illuminated by a patterned light which can be generated by grating^4^,^5^ or nonlinear patterned excitation^7^,^8^. After separating the high spatial frequency components, the subwavelength image can be resolved.

To directly get the super-resolution images of objects, various configures of so-called superlens are proposed such as negative-refractive-index medium-based perfect lenses^9^, metal-insulator stacked hyperlenses^10^,^11^, and hyperbolic metamaterials-based metalenses^12^,^13^. Although super-resolution images can be directly obtained by the superlens, they are nevertheless bounded in the near-field area of the superlens structure^14^. In the last decays, some far-field superlens configures are proposed for resolving super-resolution images from the detected far-field information of objects in free space^15^,^16^. The subwavelength structures are used to transform the evanescent waves of objects in front into propagating waves for far-field detection^15^. Also, such subwavelength structures can generate evanescent waves to illuminate objects with the scales below the resolution behind for further retrieving the super-resolution images from the scattering light or diffraction light of objects^17^,^18^. A typical far-field superlens configure is constructed with the periodic subwavelength grating^15^,^16^. The grating is used to produce a fixed phase shift in frequency domain. Therefore, the high spatial frequency information of objects could be carried to far-field area by such grating. Although the spatial resolutions of such periodic grating-based imaging systems are enhanced, we still cannot retrieve some hyperfine features of objects involved in certain spatial frequencies^15^,^19^. This is due to a critical defect of periodic subwavelength grating that it can only produce a fixed phase shift and make the frequency domain discontinuous. To overcome this shortcoming, a kind of quasi-periodic subwavelength gratings can be used to produce a quasi-continuous shift in frequency domain^19^. As the spatial frequency domain is continuous, the lost subwavelength information in the certain spatial frequency range can be carried to far-field area. Therefore, the super-resolution images may be reconstructed from the far-field detected information.

The above imaging systems are always used to observe the 1D subwavelength objects^15^,^17^,^19^. To get 2D super-resolution images by the above super-resolution microscope configures, one needs multiple-angle illuminations to objects or multiple direction detections of diffraction fields^16^,^18^. To improve such low-speed imaging process, in this paper we present a 2D Fibonacci grating-assisted microscope for far-field super-resolution imaging in free space. The evanescent waves of 2D objects can be transformed into propagating waves by the 2D Fibonacci grating, which is designed and optimized through rigorous coupled-wave analysis (RCWA) method. By measuring the far-field light intensity distributions at once, we can reconstruct the images of sample objects with a spatial resolution beyond λ/7 through the Fourier optics method^2^ and finite-difference time-domain (FDTD) numerical simulation method. Compared with the 1D Fibonacci grating-assisted microscope, which is used to extend a 1D spatial frequency axis continuous, our 2D Fibonacci grating can make frequency domain continuous in a 2D spatial frequency circular plane. Therefore, all the 2D images with different subwavelength details below the resolution can be resolved. Furthermore, we find that resolution of such imaging system in the incoherent illumination case is worse than that in coherent illumination case.

## Results

### Theoretical basis

We start our analysis from the classical Fourier optics^2^. Generally, the complex amplitude distribution of image is expressed as *g*(*x, y*) in the 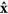-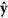 plane. In the coherent illumination case, the *g*(*x, y*) is the convolution of the complex amplitude of image *o*(*x, y*) and the point spread function (PSF) of the coherent imaging system *h*(*x, y*) as


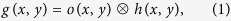


where ⊗ denotes to convolution operator^1^,^2^. Taking the Fourier transform (FT) on the equation (1), we can get *G*(*f*_*x*_, *f*_*y*_) = *O*(*f*_*x*_, *f*_*y*_) • *H*(*f*_*x*_, *f*_*y*_) in frequency domain, where *G*(*f*_*x*_, *f*_*y*_) and *O*(*f*_*x*_, *f*_*y*_) are the angular spectra of *g*(*x, y*) and *o*(*x, y*), respectively, *H*(*f*_*x*_, *f*_*y*_) is the transfer function, and *f*_*x*_, *f*_*y*_ are the spatial frequency components in the 2D Cartesian coordinate system. The intensity distribution of image is given as


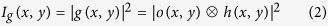


In the frequency domain, the angular spectra of *I*_*g*_(*x, y*) is written as 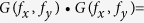

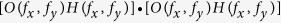
, where the symbol * represent the autocorrelation integral. The object angular spectra *O*(*f*_*x*_, *f*_*y*_) can be mathematically resolved in frequency domain from equation (1) or equation (2). Therefore, the complex amplitude distribution of object *o*(*x, y*) can be obtained by taking inverse FT on *O*(*f*_*x*_, *f*_*y*_). However, the super-resolution information of object cannot be retrieved by a conventional far-field optical imaging system because such system is diffraction limited. This means that *H*(*f*_*x*_, *f*_*y*_) = 0 when 
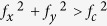
, where *f*_*c*_ is the cutoff frequency of the background medium. So the super-resolution information of the object, which is with spatial frequencies higher than *f*_*c*_, is undetectable at the imaging plane in the far field. Therefore, *g*(*x, y*) includes no super-resolution information of *o*(*x, y*) and the subwavelength details of objects are lost. Furthermore, the grating-assisted imaging systems are capable to transform the evanescent waves into propagating waves by the hyperfine structures^15^,^16^,^17^,^18^,^19^. Therefore, from the far-field light distribution *g*(*x, y*), the super-resolution images could be obtained by solving equation (1) or equation (2)^19^.

We first consider a case that a monochromatic light with wave vector **k** (*k* = 2*f*) illuminates on a 2D periodic grating. The transmission light behind such grating is with wave vector


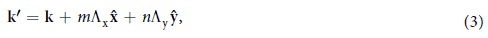


where Λ_*x*_ = 2/*p*_*x*_ andΛ_*y*_ = 2/*p*_*y*_ are the grating numbers corresponding to grating periods *p*_*x*_ and *p*_*y*_ in 2D spatial domain, the integers *m* and *n* are diffraction orders along 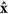 and 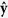 directions, respectively^2^. For far-field detecting the transmitted light in the free space, the wave vector of transmitted light should be in the range |**k**′| ≤ |**k**_0_|, in which *k*_0_ = 2*f*_0_ and *f*_0_ = 1/*λ* is the cutoff frequency in free space. From equation (3), we could get a relationship that 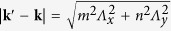. Taking out vector analysis and rewriting this expression in frequency domain, we get that the spatial frequencies of the incident light are in the range of 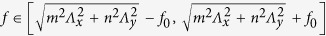.

In the following, we assume a simplified configure that such periodic grating is with equal grating periods *p*_*x*_ and *p*_*y*_ (*p* = *p*_*x*_ = *p*_*y*_). Thus, the incident light before the periodic grating could be with spatial frequencies in the range 
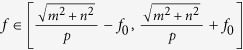
 through (m, n) diffraction order. Generally, evanescent waves are usually transformed into propagation waves through (0, 0), (±1, 0), (0, ±1), and (±1, ±1) diffraction orders, while diffraction light through higher orders than *m* ≥ 2, *n* ≥ 2 possess low diffraction efficiency for a rectangular binary grating^2^,^19^. It can also be verified by RCWA method in the following calculations. Therefore, the incident light with spatial frequencies *f*∈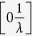  ∪  
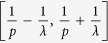
  ∪  
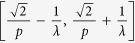
 should be detectable in the far-field area. Nevertheless, if the grating period is too small, the frequency domain will become discontinuous. For example, if period satisfies 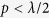, the information with spatial frequencies falling in 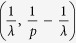, will be lost. In addition, if the period satisfies 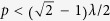, the information with spatial frequencies falling in 
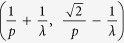
, will be lost. The lost information will lead to some subwavelength details of objects unrecoverable.

**Figure 1:**
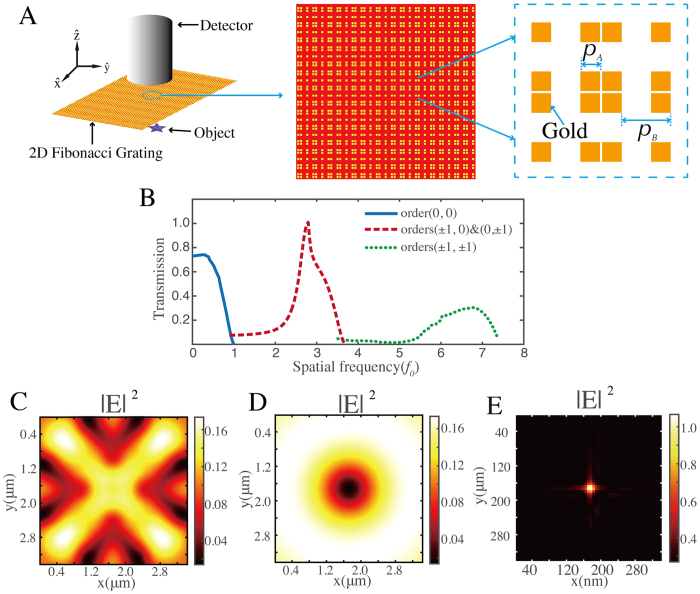
Theoretic design of the 2D Fibonacci grating-assisted super-resolution imaging system. (**A)** a Scheme of 2D Fibonacci grating. Right panel shows details of the grating. (**B)** Amplitude of transfer functions through (0, 0) order (blue solid line), higher order with minimum period *p*_*A*_ (red dash line), and higher order with long-range period 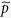 (green dot line) of the Fibonacci grating. (**C** and **D**) Calculated gray intensity distributions of PSF of the Fibonacci grating and free space, respectively. **(E)** Calculated light intensity distributions of resolved images after reconstruction process of PSF.

In 1D case, a kind of quasi-periodic Fibonacci grating was used to recovery such lost super-resolution information of 1D objects and make the frequency domain continuous in previous work^19^. In 2D case, the Fibonacci grating may also be used for super-resolution imaging the 2D objects. Such grating is consisted of blocks with subwavelength period along two orthogonal axes 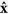 and 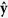, respectively (Fig. 1A). Each grating unit is subwavelength block, so the grating will show a polarization-independent response for normal incidence^20^. In each row and column, two basic units are arranged in the form of Fibonacci series as “…ABAABABAABAABABAB…” along the 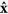 and 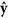 direction. Such two units are with different periods *p*_*A*_ and *p*_*B*_ (*p*_*A*_ < *p*_*B*_). The production formula of Fibonacci grating is 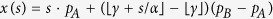, *y*(*t*) = *x*(*s*), where 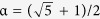, 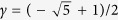, 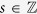, 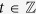, and 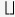 denotes to round operator. The (*x*(*s*), *y*(*t*)) gives the center position of each gating unit. Such grating structure is shown in right panel Fig. 1A. Considering the period *p* of this grating to be the minimum period *p*_*A*_, the spatial frequencies falling in 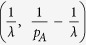, will be lost if 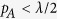 and even spatial frequencies falling in 
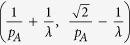
 will be lost if 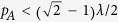. However, the Fibonacci grating has a long-range period 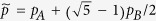. Considering the long-range period, we see that the incident frequency range can be extended to the range of 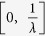  ∪  
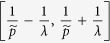
  ∪  
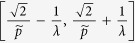
. Therefore, if the Fibonacci grating is with periods 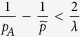, the incident frequency *f* will be extended to 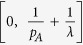 through (0, 0), (±1, 0), and (0, ±1) diffraction orders. So far, if such grating is with periods 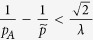, f will be extended to 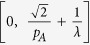 through (0, 0), (±1, 0), (0, ±1), and (±1, ±1) diffraction orders. The cutoff frequency of such imaging system is 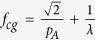. As an analytical result, the super-resolution information of objects can be carried to far field by such Fibonacci grating. The resolution of this 2D grating-assisted imaging system may exceed to 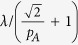. The limitation of our method is that the subwavelength grating should be placed in the near field of object. We assume that an object with spatial frequency *f* is placed with a distance *δ* along *z* direction. The intensity of the evanescent wave decrease exponentially with increasing *z* as 
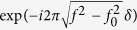
, where 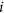 is the imaginary unit. As the distance satisfies a criterion 
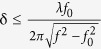
, the amplitude decreases no more than 1/e. Therefore, the evanescent waves from objects can reach grating before their vanishing.

In addtion, there are some still gaps in the 2D wave vector plane. If the periodic of grating satisfies 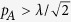, the wave vector plane could be full covered. However, there are overlap regions, meaning that frequency-shifted components are hardly separated because they contribute to different diffraction orders. To get a higher resolution, the gap inevitably exists. Some details of object fall into the forbidden wave vector regions not be resolved. One solution is to rotate the object or grating to make the frequency components fall into the transfer function plane^4^. Some other kind subwavelength structures such as quasiperiodic lattices may be capable of providing high diffraction orders because they have higher rotation symmetry.

We first use the RCWA method to calculate the diffraction efficiencies of different orders for designing the 2D Fibonacci grating with optimized geometric parameters. This grating is placed in free space with relative permittivity 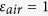. It is constructed with an array of 126 nm × 126 nm × 100 nm sized gold blocks with 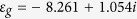. The gold also can enhance the evanescent waves of the objects in near-field area. These blocks with thickness 100 nm centered at *z* = 0 plane are arranged in the form of Fibonacci series in 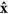-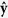 plane. The grating periods of two units are *p*_*A*_ = 140 nm and *p*_*B*_ = 140 nm along both 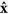 and 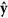 directions. The incident light wavelength is *λ *= 632 nm, which is the output wavelength of a commonly used helium-neon laser. Our RCWA calculations give the angular spectrum of the designed 2D grating. Figure 1B presents the amplitude of transfer functions from (0, 0), (±1, 0), (0, ±1), and (±1, ±1) diffraction orders. Higher diffraction orders more than *m *≥ 2, *n *≥ 2 are neglected in our cases because they show no more than 1.0e-5 efficiency and hence carry no evanescent waves to far-field area. The Fibonacci grating holds almost the same diffraction efficiencies after propagating 2.5*λ* away, which is in the far-field area. Therefore, *z* = 2.5*λ* is chosen as the imaging plane in the following discussions. By considering the minimum period *p*_*A*_ = 140 nm and (0, 0), (±1, 0), (0, ±1), and (±1, ±1) diffraction orders, we can find that the incident spatial frequency domain is extended to f ∈[0, *f*_0_] ∪ [3.51*f*_0_, 7.38*f*_0_] (Fig. 1B, blue solid line and green dot line). For a long-range period 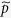 = 335 nm, the spatial frequency domain of incident light is extended to [0, 3.67*f*_0_] (Fig. 1B, blue solid line and red dash line), which covers the missing range (f0, 3.51*f*_0_). Therefore, through nine diffraction orders, the incident light in the frequency range [0, 7.38*f*_0_] can be transformed into propagating waves. The evanescent waves with spatial frequencies falling in [*f*_0_, 7.38*f*_0_] can be carried to the far field by this 2D Fibonacci grating. So the resolution of such imaging system exceeds *λ*/7.38≈86 nm. Figure 1C shows the intensity distribution 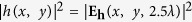 of PSF at the imaging plane by considering (0, 0), (±1, 0), (0, ±1), and (±1, ±1) diffraction orders. For comparison, we also calculate the PSF at the imaging plane in free space shown in Fig. 1D. The PSF shown in Fig. 1C and itself are used in deconvolution process. Figure 1E presents the gray distribution of retrieved image. This result describes the resolution of the 2D Fibonacci grating system.

### Simulation scheme

**Figure 2:**
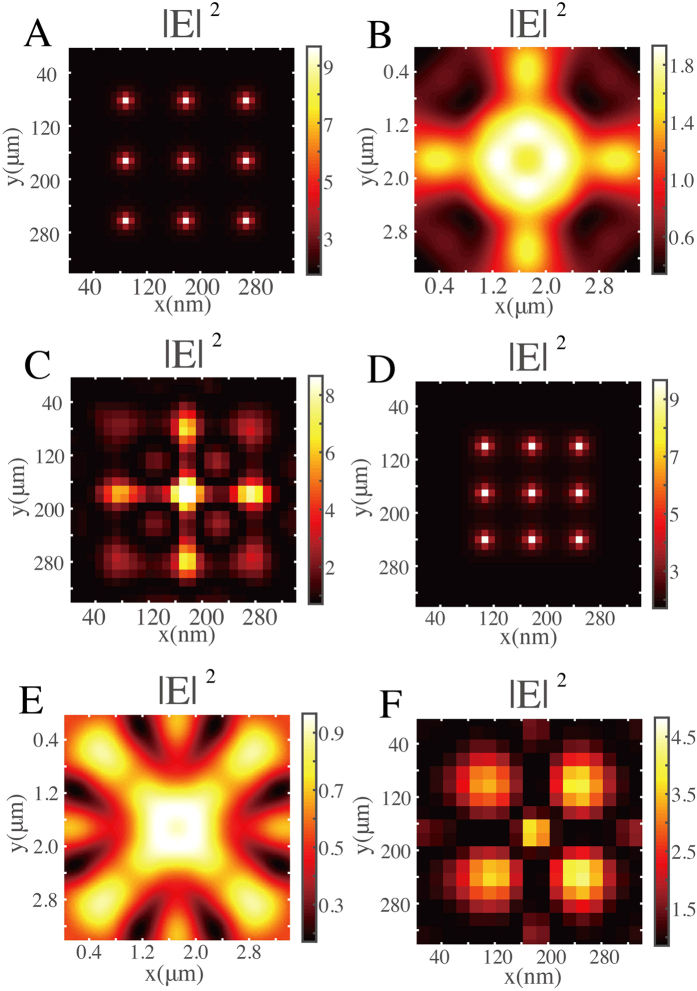
Simulation results of super-resolution imaging by the 2D Fibonacci grating in the coherent illumination case. (**A** and **D)** Gray intensity distributions of 3 × 3-point-array object spaced by 90 nm and 70 nm, respectively. (**B** and **E)** Simulated raw images in the case of coherent light illumination at wavelength of 632 nm of A and D, respectively. (**C** and **F)** Calculated light intensity distributions of resolved images after reconstruction process of A and D, respectively.

Now, by using FDTD method, we simulate the transmission light intensity distribution 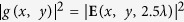 at the imaging plane *z* = 2.5*λ* when a light with *λ* = 632 nm passes through a subwavelength object and the Fibonacci grating (centered at plane *z* = 0) behind the object placed at object plane *z* = −60 nm (Fig. 2A). The distance between the object and grating is *δ* = 10 nm, which meets the distance criterion discussed above. The illuminating light is a TEM-polarized plane light (magnetic field **H** and electronic field E perpendicular to the 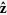 direction) normal to the grating. As an example, we chose a 3 × 3-point-array object spaced by 90 nm as a sample to be reconstructed. These nine points are arranged as (−90 nm, −90 nm), (−90 nm, 0), (−90 nm, 90 nm), (0, −90 nm), (0, 0), (0, 90 nm), (90 nm, −90 nm), (90 nm, 0), and (90 nm, 90 nm). Such an object contains the super-resolution information between 90 nm~255 nm, such as some two-point objects spaced by 90 nm, 127 nm, 180 nm, 201 nm, and 255 nm, respectively. In FDTD simulations, the grid is 10 nm along the 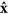, 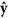, and 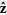 directions, so each point object is a 10 nm × 10 nm square. The intensity of illuminating light is set as 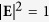. The convolutional perfect matched layer is employed as the absorbing condition in 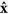, 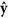, and 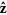 directions. The gray intensity distribution of the object in the near-field area at plane *z* = −60 nm is shown in Fig. 2A. Figure 2B shows the simulated transmission light intensity distribution at image plane *z* = 2.5*λ* (raw image) through the object and the Fibonacci grating. For comparison, we also simulate the raw image of such object only in free space, which is the same as shown in Fig. 1D. Obviously, we see that the Fibonacci grating changes the object light. The PSF 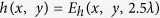 of this imaging system is calculated by using the RCWA method. The intensity distribution of PSF is shown in Fig. 1C. The images of objects can be reconstructed from the transmission light intensity distributions at plane *z* = 2.5*λ* by solving the equation (2). With the help of deconvolution calculations based on time-reversal transmission matrix method^19^ (see the detailed steps in Methods), the *o*(*x, y*) at object plane *z* = −60 nm can be obtained.

To deduce the deconvolution errors by the asymmetric field distributions, the object to be observed is placed at the center of the grating. The raw image shown in Fig. 2B and PSF shown in Fig. 1C are used in deconvolution calculation process. Figure 2C presents the gray distribution of retrieved image. We can see a 3 × 3-point-array object spaced with 90 nm, which is approximately *in situ* to the original object as shown in Fig. 2A. In the deconvolution process, the regularization parameter^21^ is a constant added onto the intensity matrix of transfer function *H*(*f*_*x*_, *f*_*y*_). The transfer function is related to the diffraction efficiencies. So if the efficiencies of (±1, ±1) diffraction orders are much smaller than the efficiencies of (0, 0), (±1, 0), and (0, ±1) diffraction orders, some numerical error will be introduced into the reconstruction procedure and interpret the results. So we see that the amplitudes of each point in Fig. 2C are with a little difference.

For comparison, we replace the sample object to another 3 × 3-point-array object spaced by 70 nm. Such super-resolution information is beyond the resolution of our designed Fibonacci grating (86 nm). Some two-point objects spaced by 99 nm, 140 nm, 198 nm below the resolution can be found in the sample object. The light intensity distribution of the original object in the near-field area at plane *z* = −60 nm is shown in Fig. 2D. Figure 2E shows the gray distribution of raw image at imaging plane *z* = 2.5*λ*. Taking the raw image shown in Fig. 2E and PSF shown in Fig. 1C into the image retrieving process, we get the retrieved image shown in Fig. 2F. We see that the sample object cannot be correctly resolved. However, we can obtain a five-point image, which contains a 2 × 2-points array spaced by 140 nm and a center point with 99 nm to the other four points around. Because these subwavelength details below the resolution of such Fibonacci grating can still be observed. However, in the 2D wave vector plane in the above discussions, there are some gaps. If the 3 × 3 90 nm array is turned by 30° degrees or by 45°, some high frequency Fourier components of object fall into the gaps. So we cannot reconstruct a correct image of such example.

In the above discussions, the imaging process is considered in a coherent illumination case. Furthermore, we investigate the super-resolution imaging effect by the above Fibonacci grating-assisted system in an incoherent illumination case. In the incoherent illumination case, the image intensity is given by the convolution equation


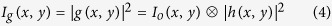


where 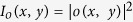 is intensity distribution of object light. On the other hand, in the coherent illumination case, the image process is expressed in equation (2).

**Figure 3:**
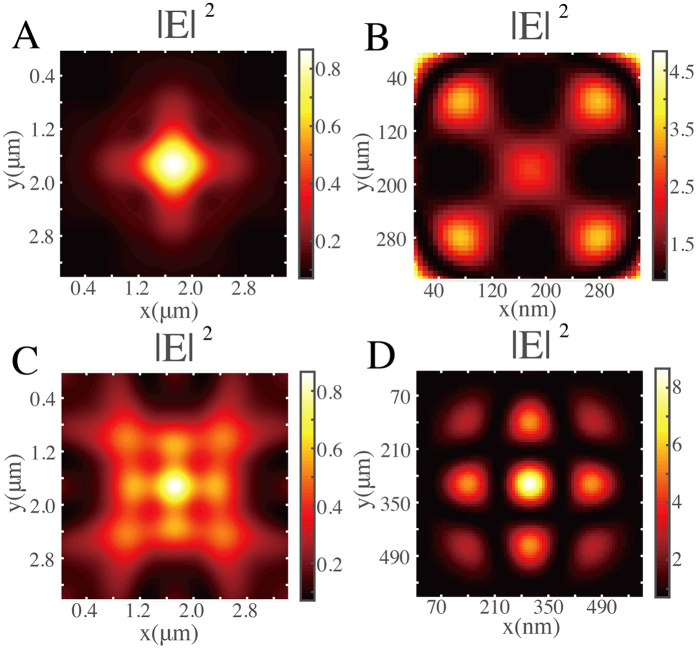
Simulation results of super-resolution imaging by the 2D Fibonacci grating in the incoherent illumination case. (**A** and **C)** Simulated joint raw image of 3 × 3-point-array object spaced by 90 nm and 170 nm, respectively. (**B** and **D)** Calculated light intensity distributions of resolved images after reconstruction process of A and D, respectively.

Next, we also chose a 3 × 3-point-array object spaced by 90 nm as a sample to be reconstructed. To simulate the raw image in the incoherent illumination case, this sample object is divided into nine single-point objects. Each single-point object is illuminated by the incident light with wavelength 632 nm. Their intensity distributions at image plane *z* = 2.5*λ* are simulated individually. Then the joint raw image of incoherent illumination case is gotten by summing all the intensity distributions of single-point objects. Figure 3A presents the intensity distribution of the raw image. In the image retrieving process, the deconvolution calculation is used for solving equation (4). The raw image shown in Fig. 3A and PSF shown in Fig. 1C are used in deconvolution calculation process. The image intensity distributions are used for retrieving image instead of image amplitude distributions. Figure 3B presents the intensity distribution of retrieved image. However, we cannot see a correct resolved image of the sample object. For comparison, we also replace the sample object to another 3 × 3-point-array object spaced by 170 nm, which can also be resolved in the coherent illumination case. Figure 3C shows the intensity distribution of raw image at imaging plane. Taking the raw image shown in Fig. 3C and PSF intensity distribution shown in Fig. 1C into the image retrieving process, we get the retrieved image shown in Fig. 3D. We can get a 3 × 3-point-array image spaced with 170 nm, which is the same as the original object. There is no meaningful criterion to specify that incoherent illumination or coherent illumination will yield a “better” resolution^2^. However, from the above simulation results, we can obtain a result that the coherent illumination case yields a better resolution than incoherent illumination case by our designed 2D Fibonacci grating.

In our discussed imaging system, the frequency spectra of objects are transformed to far-field area by the subwavelength grating. In the incoherent case, the frequency spectra of the object intensity are written as 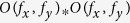. Thus, the frequency range of object intensity in the incoherent case is twice of the range of *O*(*f*_*x*_, *f*_*y*_) in the coherent case. However, the cutoff frequency of the grating is still *f*_*cg*_ from the above analysis of equation (3). The object intensity with spatial frequencies higher than *f*_*cg*_ cannot be transformed to far-field area in incoherent illumination case. So the spatial frequency range of object amplitude are in the range of [0, *f*_*cg*_/2], meaning that the objects are imaged with a better resolution in coherent case than that in incoherent case. The resolution of incoherent case is twice of that of coherent case, which equals to 172 nm by the Fibonacci grating. This analytical result is well consistent with above simulation result.

## Discussion

To conclude, we have demonstrated that a Fibonacci 2D grating can be used to transform evanescent waves into propagation waves for far-field super-resolution imaging. By detecting the far-field light distributions of light through objects in front of the grating in free space, we retrieved the images of objects beyond λ/7 spatial resolution. The advantage of this method is that all the super-resolution details below the resolution of this system can be resolved from the far-field detected information at one time. We also find that coherent illumination case yields a better resolution than incoherent illumination case by such imaging system. This idea of evanescent transformation is useful for biomedical imaging applications and nanotechnology.

## Methods

### Deconvolution procedure

(1) Estimation of *G*( *f*_*x*_, *f*_*y*_ ) and *H*( *f*_*x*_, *f*_*y*_ ) by taking Fourier transformation on *g*(*x, y*) and *h*(*x, y*), respectively:


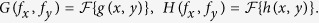


(2) Calculation of the time-reversal (conjugated) matrix of *H*( *f*_*x*_, *f*_*y*_ ) as H*( *f*_*x*_, *f*_*y*_ ).

(3) Retrieval of *O*( *f*_*x*_, *f*_*y*_ ):


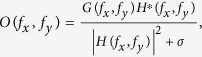


where *σ* is a regularization parameter near the numerical precision limit^21^.

(4) Estimation of *o*(*x, y*) by taking inverse Fourier transformation on 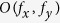:


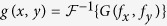
.

## Additional Information

**How to cite this article**: Wu, K. and Wang, G. P. Two-dimensional Fibonacci grating for far-field super-resolution imaging. *Sci. Rep.* **6**, 38651; doi: 10.1038/srep38651 (2016).

**Publisher's note:** Springer Nature remains neutral with regard to jurisdictional claims in published maps and institutional affiliations.
